# Development and assessment of a vaccine administration training course for medical students

**DOI:** 10.1186/s12909-023-04299-w

**Published:** 2023-05-25

**Authors:** Hirohisa Fujikawa, Daisuke Son, Hiroko Mori, Satoshi Kondo, Shoko Horita, Masashi Izumiya, Masato Eto

**Affiliations:** 1 Center for General Medicine Education, School of Medicine, Keio University, 35 Shinanomachi, Shinjuku-ku, Tokyo, 160-8582 Japan; 2 Department of Medical Education Studies, International Research Center for Medical Education, Graduate School of Medicine, The University of Tokyo, Bunkyo-ku, Tokyo, Japan; 3 Department of Community-based Family Medicine, Faculty of Medicine, Tottori University, Yonago, Tottori Japan; 4 General Education Center, The University of Tokyo Hospital, Bunkyo-ku, Tokyo, Japan; 5 Center for Medical Education and Career Development, Graduate School of Medicine, University of Toyama, Toyama, Toyama Japan; 6 Department of Medical Education, School of Medicine, Teikyo University, Itabashi-ku, Tokyo, Japan

**Keywords:** Vaccination, Vaccine administration, Confidence, Skill, Near-peer learning, Medical students, Professional competence, Professional identity development, Procedural learning

## Abstract

**Background:**

Vaccine administration skills are very important for physicians, especially in the era of global pandemics. However, medical students have reported that practical sessions to develop these skills are insufficient. Therefore, the aim of our study was to develop a vaccination training course for medical students. We also examined its educational effectiveness.

**Methods:**

5th- and 6th-year medical students at the University of Tokyo were recruited to attend the vaccine administration training course in 2021. These students were our study participants. Our course consisted of an orientation part, which included a lecture on the indications, adverse events, and vaccination techniques of flu vaccines and practice on a simulator, and a main part in which the staff of the University of Tokyo Hospital were actually vaccinated. Before and after the main part of the course, study participants completed an online questionnaire that assessed their confidence in vaccine administration technique through a five-point Likert scale. We also surveyed their feedback about the course content and process. At the beginning and end of the main part, their technical competence in vaccination was assessed by two independent doctors. These doctors used a validated checklist scale (ranging from 16 to 80) and a global rating scale (ranging from 0 to 10). We used their mean scores for analysis. The quantitative data were analyzed through the Wilcoxon signed-rank test. For the qualitative data of the questionnaire, thematic analysis was conducted.

**Results:**

All 48 course participants participated in our study. Participants’ confidence in vaccination technique (*Z* = -5.244, p < 0.05) and vaccination skill significantly improved (checklist rating: *Z* = -5.852, p < 0.05; global rating: *Z* = -5.868, p < 0.05). All participants rated the course as, “overall educational.” Our thematic analysis identified four emerging themes: interest in medical procedures, efficacy of supervision and feedback, efficacy of “near-peer” learning, and very instructive course.

**Conclusions:**

In our study, we developed a vaccine administration course for medical students, assessed their vaccination techniques and confidence in those techniques, and investigated their perceptions of the course. Students’ vaccination skills and confidence improved significantly after the course, and they positively evaluated the course based on a variety of factors. Our course will be effective in educating medical students about vaccination techniques.

**Supplementary Information:**

The online version contains supplementary material available at 10.1186/s12909-023-04299-w.

## Background

Infectious diseases have long been a major public health concern worldwide [1]. In 2019, the spread of the coronavirus disease (COVID-19) became a major threat [2] and by the end of 2021, more than 250 million people, globally, had been infected by it [3]. An important component of the public health response to the pandemic was vaccines [4].

Vaccines are a crucial tool in the fight against infectious diseases [5]. Ever since Edward Jenner developed the first vaccine in the 18th century [6], various vaccines have been developed and used to save millions of lives [4]. As of 2022, in Japan, routine vaccines include vaccines for hepatitis B, *Haemophilus influenzae* type b, pneumococcus, rotavirus, diphtheria, pertussis, tetanus, polio, tuberculosis, measles, rubella, varicella, Japanese encephalitis, and human papillomavirus. The vaccines for influenza and mumps are included in voluntary vaccines. And since the 2020s, the COVID-19 vaccine has also been used to reduce death, severe cases, symptomatic cases, and infections worldwide [2, 7]. Thus, the knowledge about vaccination techniques are essential for physicians, and it is important that they are trained in these techniques at the earliest [8].

Nevertheless, medical students lack experience in most basic medical procedures and rate themselves as being unable to perform the procedures independently, even just prior to entering residency [9, 10]. In addition, according to a survey study of incoming first-year residents in the United States, they report a lack of confidence in their ability to perform the common basic procedures [11].

In Japan, the standard undergraduate medical education program lasts six years [12]. In the first and second years, students receive preclinical education. In the third and fourth years, they study clinical medicine, including infectious disease medicine. During the third and fourth years, students essentially attend classroom lectures, including infectious diseases, with little or no exposure to the medical field or the practice of medical procedures. Finally, in the fifth through sixth years, medical students are placed in clinical practice. In Japan, a shift from the traditional “observational” to “participatory” clinical clerkship, or more active participation of medical students in clinical practice, has been touted in recent years; however, in practice, the transition has not been successful. For example, approximately 20% of medical students performed subcutaneous, intradermal, and intramuscular injections under the guidance and supervision of a supervising physician, yet fewer than 5% were confident enough to perform the procedure independently [13]. Thus, even in Japan, medical students’ opportunities and confidence in vaccination techniques are inadequate. In Japan, vaccination procedures are frequently performed by physicians, not by other health professionals. If there is insufficient undergraduate training in vaccine administration, then after entering residency, one is suddenly put into practice, which may lead to patient safety issues. Therefore, it is critical to educate medical students about vaccination during their undergraduate years.

To the best of our knowledge, no study has examined the educational benefits of a course in which medical students vaccinate real people. In the previous study conducted in Australia, the authors examined the educational effectiveness of a vaccination program for pharmacy students. However, in this Australian program, the target to be vaccinated was a mannequin [14]. Although simulation-based education has recently evolved [15, 16], performing procedures on live patients is considered an irreplaceable experience [9]. The University of Tokyo has been conducting a vaccination training course for medical students since 2019. In this course, fifth-year (pre-final year) and sixth-year (final year) medical students administer vaccines to staff of the University of Tokyo Hospital. When we began this course in 2019, course participants’ vaccination techniques appeared to have improved dramatically, and medical students’ perceptions of the course appeared to be good, but this could not be assessed in 2019–2020. Hence, we wanted to examine the educational effectiveness of this course in 2021.

## Methods

### The course

In October-November 2021, a vaccine administration training course was held for medical students at the University of Tokyo. A review of the literature on immunization training of healthcare students revealed a variety of pedagogical strategies used to promote understanding of vaccination techniques and to improve vaccination skills [14, 17]. In our course, we held an orientation part and a main part. The course details are summarized in Table 1.

**Table 1:** The details of the vaccine administration training course for medical students at the University of Tokyo in 2021

Orientation part (one month prior to the main part)	Main part
• Lecture • Practice using a simulator ¬ The supervising physicians provides guidance as needed	• Vaccinations for hospital staff ¬ Split into groups of 2–3 ¬ Mutual help and teaching when necessary ¬ The supervising physicians provided the required guidance • Reflection

First, in October 2021, we held an orientation part for the participants. In Japan, the influenza vaccine is customarily administered subcutaneously. Thus, in the orientation part, we focused on subcutaneous injections among injection techniques. We also discussed flu vaccine indications, effectiveness, potential complications, and troubleshooting. After these explanations, course participants practiced subcutaneous vaccination using a simulator. During the practice, the supervising physicians stood by to observe and provide guidance as needed.

Then, in November 2021, a four-day main part was held. During the four-day part, each day had one morning session (9:30–11:30) and one afternoon session (13:30–15:30) and a total of eight instruction sessions were provided. The 48 medical students who volunteered to participate in the course attended only one session each and administered the flu vaccine subcutaneously to hospital staff. There were 5–7 participants per session. Participants were divided into groups of two or three, with one member acting as the inoculator and the rest acting as support, switching roles as appropriate, to ensure that vaccination opportunities were generally equal. For the safety of the vaccine recipients and the learning effectiveness of the participants, the supervising physicians was right next to the participants, watching over them. The physicians provided the required guidance to the participants. The medical students helped each other with vaccination techniques and taught each other as needed. We predicted that interaction among medical students would certainly be beneficial, as they rarely interact with each other in their daily clinical practice. After each session, the medical students and the supervising physicians gathered for reflection for further learning in the future.

### Participants

The faculty of the department of the Medical Education Studies (MI and ME) sent an email to all the 230 fifth- and sixth- year medical students, inviting them to participate in this course. Given the capacity of the vaccination site, we decided to recruit approximately 50 students on a first-come, first-served basis. We received a response from 48 students. HF, a PhD student in the department of Medical Education Studies, then sent an email to these 48 course participants inviting them to participate in the study. Each student was informed that their participation in the course and study was voluntary and that there would be no disadvantages or grade consequences for participation or non-participation. Finally, all 48 course participants also agreed to participate in the study.

### Measures

A self-report online questionnaire was distributed to the study participants before and after the main part (Table 2). We used SurveyMonkey to distribute our questionnaire and collect responses. There were no instruments available in Japanese for use in assessing courses such as the one we developed this time. Therefore, the 1st author (HF) developed an instrument to assess this course, and the contents were discussed and agreed upon by the team of all authors. In addition to closed-questions related to vaccination, daily clinical clerkship, and the course, open-ended questions were also used to elicit the participants’ impressions and feedback of the course [18].

**Table 2:** Details of the questionnaire before and after the main part of the vaccine administration training course

Before	After
• Demographic questions • Confidence in vaccination technique (1 = Unable to perform vaccine administration to 5 = Performs vaccine administration easily and with fluidity) • Motivation towards daily clinical clerkship (0 to 10 (worst to best))	• Confidence in vaccination technique (1 = Unable to perform vaccine administration to 5 = Performs vaccine administration easily and with fluidity) • Motivation towards daily clinical clerkship (0 to 10 (worst to best)) • Feedback survey (1 = Strongly disagree to 5 = Strongly agree) ¬ Feedback from supervisors was useful ¬ Feedback from other medical students was useful ¬ The lecture given during orientation was useful ¬ Prior practice in the simulator was useful ¬ The course was suitable for my level ¬ The course will be useful in my future career as a doctor ¬ I would like to recommend this course to other medical students ¬ Overall, the course was educationally worthwhile • Open-ended questions ¬ Additional comments on this course ¬ Additional comments on factors regarding this course that you would like to see continued in the future

In the questionnaire, participants were asked about their confidence in vaccine administration before and after the course. It was rated on a five-point Likert scale (1 = Unable to perform vaccine administration, 2 = Performs vaccine administration with much help, 3 = Performs vaccine administration with some help, 4 = Performs vaccine administration with minimal help, and 5 = Performs vaccine administration easily and with fluidity). The questionnaire also asked participants to rate their motivation towards daily clinical clerkship using a global rating scale of 0 to 10 (worst to best) before and after the course. In the field of medical education research, the utility of global rating scales has been proposed because they are better at capturing subtle nuances [19–21]. Motivation is a multifaceted concept, and its nuances may be better captured by global rating scales. Therefore, we decided to use the global rating scale.

The technical competence of the medical students, to administer vaccination, were assessed at the beginning and end of the main part by two independent physicians. All end-of-session assessments were conducted without reference to the assessment sheet at the beginning of the session. To assess the skills, we used an existing instrument, which was developed through a modified Delphi process [22]. The Delphi is an established process for consensus development among various stakeholders [23–25]. A validation study had verified the inter-rater reliability and concurrent validity of this instrument [22]. The instrument comprised of 19 items. Each item was rated on a 5-point Likert scale (1 = Unable to perform step, 2 = Performs step with much help, 3 = Performs step with some help, 4 = Performs step with minimal help, and 5 = Performs step easily and with fluidity). In this course, since pre-vaccination screening, informed consent, and documentation were done by physicians, and not medical students, we decided to cover a total of 16 items, and the corresponding 3 items in the checklist were marked as “not applicable” (Additional file 1). We used the average of the total scores measured by the two physicians, which ranged from 16 to 80, with higher scores indicating greater competency in vaccine administration. On the other hand, previous studies had indicated that the use of a global rating was superior to the use of a checklist in assessing technical competence [19, 26]. Therefore, in our study too we used global rating that we used in our previous study [22] and asked the two physicians to rate the participants accordingly (“Using any number from 0 to 10, in which 0 is the worst vaccinator possible and 10 is the best vaccinator possible, what number would you use to rate this vaccinator?”). An average of their response score was used for analysis. The number of vaccinations given by each student was counted by the supervising physicians present.

### Ethical considerations

All the participants provided written consent to participate in this study. We received ethics approval from the Institutional Review Board of the University of Tokyo (2020364NI).

### Statistical analyses

Quantitative data were assessed for normality and described using a non-parametric methodology. A Wilcoxon signed rank test was used to determine the significance of difference before and after the course. p < 0.05 was considered significant. All statistical analyses of quantitative data were completed using SPSS 27.0 (IBM Japan; Tokyo, Japan). For open-ended survey responses, we conducted a thematic analysis with an inductive approach [27]. First, HF generated initial codes. HF and DS iteratively discussed and reviewed the codes. HM, SK, SH, MI, and ME checked the results. Finally, all authors discussed the results and reached a consensus.

## Results

### Descriptive statistics

All 48 course participants were included in our study. The participants had little experience in vaccination procedures before participating in the course. During the main part of the course, they vaccinated 21.38 (3.98) [16–30] (mean (standard deviation) [range]) hospital staff. Table 3 summarizes the descriptive statistics.

**Table 3:** Descriptive statistics of the participants

Gender, N (%)	
Female Male Unanswered	13 (27.1) 34 (70.8) 1 (2.1)
Academic level, N (%)	
Fifth-year Sixth-year	36 (75.0) 12 (25.0)
Number of medical procedures experienced, N (%)	
0 1 2 3–5 6–10 > 10	33 (68.8) 8 (16.7) 1 (2.1) 0 (0) 3 (6.3) 3 (6.3)
Number of vaccine administration experienced, N (%)	
0 1 2 3–5 6–10 > 10	39 (81.3) 3 (6.3) 0 (0) 0 (0) 4 (8.3) 2 (4.2)

### Pre/post analysis of confidence in vaccination procedures, motivation towards clinical clerkship, and competence of vaccination procedures

Students felt significantly more confident after the course (*Z* = -5.244, p < 0.05). We found significant improvement in motivation towards clinical clerkship after the course (*Z* = -3.363, p < 0.05). Despite the presence of ceiling effect, both the checklist and global ratings showed that the technical competence of vaccine administration significantly improved during the course (*Z* = -5.852, p < 0.05, and *Z* = -5.868, p < 0.05, respectively) (Table 4).

**Table 4:** Wilcoxon signed-rank test analysis of confidence in vaccination procedures, motivation towards clinical clerkship, and competence of vaccination procedures

	Pre (Median (IQR))	Post (Median (IQR))	Z
Confidence in vaccine administration	3.00 (3.00-3.75)	4.00 (4.00–5.00)	-5.244*
Motivation towards clinical clerkship	7.00 (6.25-8.00)	8.00 (7.00–9.00)	-3.363*
Competence of vaccine administration (checklist)	78.000 (75.875–78.500)	80.000 (80.000–80.000)	-5.852*
Competence of vaccine administration (global rating)	8.000 (7.500–8.500)	10.000 (10.000–10.000)	-5.868*

Abbreviations: IQR Interquartile range

Note: Confidence in vaccine administration ranges from 1 to 5, with higher scores indicating more confidence in vaccine administration. Motivation towards clinical clerkship ranges from 0 to 10, with higher scores indicating more motivated. Competence of vaccine administration (checklist) ranges from 16 to 80, with higher scores indicating more competent in vaccine administration. Competence of vaccine administration (global rating) ranges from 0 to 10, with higher scores indicating more competent in vaccine administration. * indicates p < 0.05

### Feedback survey

Overall, the medical students gave high scores for the course. Table 5 summarizes the results of the quantitative feedback survey.

**Table 5:** Feedback survey

	Strongly disagree	Disagree	Neither agree nor disagree	Agree	Strongly agree
	N (%)	N (%)	N (%)	N (%)	N (%)
Feedback from supervisors was useful.	0 (0)	1 (2.1)	0 (0)	20 (41.7)	27 (56.3)
Feedback from other medical students was useful.	0 (0)	2 (4.2)	5 (10.4)	21 (43.8)	20 (41.7)
The lecture given during orientation was useful.	0 (0)	1 (2.1)	7 (14.6)	17 (35.4)	23 (47.9)
Prior practice in the simulator was useful.	0 (0)	0 (0)	7 (14.6)	17 (35.4)	24 (50.0)
The course was suitable for my level.	0 (0)	0 (0)	0 (0)	15 (31.2)	33 (68.8)
The course will be useful in my future career as a doctor.	0 (0)	0 (0)	0 (0)	13 (27.1)	35 (72.9)
I would like to recommend this course to other medical students.	0 (0)	0 (0)	0 (0)	17 (35.4)	31 (64.6)
Overall, the course was educationally worthwhile.	0 (0)	0 (0)	0 (0)	8 (16.7)	40 (83.3)

A total of 21 responses were obtained to the open-ended questions. Participants’ qualitative feedback was clustered into four emergent themes: (1) Interest in medical procedures; (2) Efficacy of supervision and feedback by supervising physicians; (3) Efficacy of “near-peer” learning; and (4) Very instructive course.

#### Theme 1: interest in medical procedures

Participants felt that their interest in medical procedures had increased, as they performed them many times during this course.

*“Having performed medical procedures, I became interested in them.” (Participant 13, 5th medical student)*.

#### Theme 2: efficacy of supervision and feedback by supervising physicians

Medical students felt comfortable performing vaccination procedures during the course because these were performed under the supervision of the physicians.

*“The supervising physicians were always watching us [during the course], so I was able to work on the vaccination procedures with peace of mind.” (Participant 10, 5th medical student)*.

Participants felt that feedback from supervising physicians was effective.

*“[If this course is offered again next year,] I hope that the faculty will continue to provide feedback on the vaccine administration procedures.” (Participant 10, 5th medical student)*.

*“I appreciated the feedback [from the faculty] during the course.” (Participant 13, 5th medical student)*.

#### Theme 3: efficacy of “near-peer” learning

In this course, fifth- and sixth-year medical students, who do not normally interact with each other in clinical practice, had to work as a team to administer vaccines. Sixth-year medical students occasionally gave advice to fifth-year medical students on vaccination techniques. One participant described this “near-peer” learning as follows:

*“Having fifth- and sixth-year medical students working on the same team was very effective for our learning.” (Participant 32, 6th medical student)*.

#### Theme 4: very instructive course

Overall, medical students felt that the course was very instructive and provided a valuable learning experience.

*“I learned a lot from this course” (Participant 26, 6th medical student)*.

*“I became better at vaccination techniques when I actually experienced them in the field rather than simulating them over and over again.” (Participant 11, 5th medical student)*.

## Discussion

Although the vaccine administration technique is crucial for physicians, especially in this era of a global pandemic, there has been insufficient education with regard to it. Further, the competency of medical students on vaccination techniques and the existence of relevant educational courses for the technique have never been examined. In the present study, we developed an intensive vaccination course for medical students and verified its educational effectiveness. The findings of this study will be beneficial to the future educators of infectious diseases.

In our course, most of the medical students indicated that supervisory feedback was effective. According to previous studies, simply performing medical procedures without feedback may have improved the trainees’ confidence, but not their skills [28]. Therefore, direct observations and immediate feedback from experts are crucial for the development of medical procedural skills [29, 30]. The results of the present study support these findings.

The results of this study also showed that many students reported the effectiveness of feedback. In particular, the results of the qualitative data indicate educational benefits in pairing fifth-year students with sixth-year students. Past medical education studies have also demonstrated the effectiveness of “near-peer teaching” (the teaching of junior students by students who are seniors by one or more years of education) [31]. Bulte and colleagues found that students consider near-peer teachers to be more cognitively closer than teachers [32]. Near-peer teachers may be in a better position to understand the problems faced by students and to explain difficult concepts at an appropriate level [33]. Besides, near-peer teaching experience may enhance the teacher’s own learning and motivate them [34]. Thus, near-peer teaching seems to be beneficial for both teachers and students, and incorporating it into the vaccination training course can be advantageous.

In the current undergraduate medical education, medical students have few opportunities to perform most basic clinical procedures and lack the confidence and ability to perform them independently. In recent years, the potential usefulness of simulation-based education has become apparent [35]. Besides, performing medical procedures on real patients can be a stressful experience for any practitioner, but especially for novices [36]. However, the experiential nuances that come from dealing with live patients remain invaluable [9]. Overall, vaccine administration is a safe clinical procedure, with a low incidence of complications and low severity of complications [37]. This makes it easier for both patients and medical students to accept that the procedure would be performed by medical students. Therefore, in future undergraduate medical education, medical students should be trained to administer vaccinations to live patients. Our course model could be exported to other institutions, especially in Japan, and thus contribute to the undergraduate medical education in the future.

There are some limitations to our findings. First, there is a possibility of sample bias (i.e., students who participated could have been more interested in vaccination skills). If the course is formally incorporated into future undergraduate medical education curricula, examining its educational effects further will enrich the findings of such studies. Second, this is a single-center study. Further studies that can include medical students from multiple medical schools are required. Third, we were only able to evaluate the short-term outcome after the course, further verification is needed to evaluate its long-term outcome. Fourth, vaccination techniques were evaluated only from the perspective of physicians. In future studies, it would be helpful to have opinions from interprofessional healthcare teams and patients to deepen our knowledge. Fifth, it was the hospital staff who received the vaccine, not the general public in the strict sense of the word. According to a previous research, patients are reluctant to have medical students perform clinical procedures on them [38]. Therefore, caution may be needed if this course uses genuine patients rather than hospital staff. Sixth, the scales used to measure our course were originally developed by the authors and have not been properly validated. Further validation of the scales would be needed in future studies.

## Conclusions

The vaccination training course, which we had developed for medical students, significantly improved their confidence and competence in the vaccination techniques. All participants rated the course “overall, educational.” Four themes emerged from participants’ qualitative feedback: interest in medical practice, effectiveness of supervision and feedback by supervising physicians, effectiveness of “near-peer” learning, and very instructive course. This course would be effective in the education of medical students about vaccine administration.

## Electronic supplementary material

Below is the link to the electronic supplementary material.

Supplementary Material 1

## Data Availability

The datasets generated and/or analyzed during the current study are available from the corresponding author on reasonable request.
