# Supplementary material for: Development and assessment of a vaccine administration training course for medical students
Source: BMC Med Educ. 2023 May 25;23:385. doi: 10.1186/s12909-023-04299-w (PMC10211302; doi:10.1186/s12909-023-04299-w)
Supplement: Supplementary file 1 — Supplementary Material 1 [file 12909_2023_4299_MOESM1_ESM.docx]

Vaccine performance assessment checklist [22]

The following items are rated on five-point scale (1 = unable to perform step, 2 = performs step with much help, 3 = performs step with some help, 4 = performs step with minimal help, and 5 = performs step easily and with fluidity) or N/A (not applicable).

1. Introduces self to patient

2. Checks patient's identity

3. Checks that indication exists for the vaccine administration

4. Obtains consent for procedure from patient or representative

5. Prepares and checks for necessary equipment

6. Performs hand hygiene

7. Positions the patient so that his/her arms are in the proper position

8. Identifies proper site of needle insertion*

9. Checks for alcohol sensitivity**

10. Disinfects the puncture site with an antiseptic (alcohol or alcohol free wipes)**

11. Informs the patient that the skin will be punctured

12. Punctures*

13. Ensures that there is no numbness after the puncture

14. Injects the vaccine

15. Removes needle

16. Disposes of used items separately in the trash according to whether they are sharp or infectious

17. Documents

18. Communicates with medical and administrative staff as appropriate throughout the entire process

19. Communicates with the patient as appropriate throughout the entire process

*For details, please refer to the guidelines of each country and facility. The procedures commonly performed in Japan are as follows:

- Subcutaneous injection: Punctures in the lower third of the midline of the posterior side of the upper arm at a 45° angle to the skin
- Intramuscular injection: Punctures in the intersection of the anteroposterior axillary line (the line connecting the upper end of the anterior axillary line with that of the posterior axillary line) and the vertical line from the mid-acromion or 2–3 finger breadths below the mid-acromion at a 90° angle to the skin

**In a strict sense, if the skin is visibly clean, disinfection is not required medically. However, it is customary in some countries and facilities. Please follow the guidelines of each country and facility.

Note: In the present study, “N/A” was selected for Q3, Q4, and Q17 because these were done by physicians.
